# Indirect Fistula: A New Terminology for Cerebrospinal Fluid Fistula With Different ‘Apparent Origin’ and ‘Real Origin’

**DOI:** 10.7759/cureus.60250

**Published:** 2024-05-14

**Authors:** Enrique Caro-Osorio, Carlos D Acevedo-Castillo, Azalea Garza-Baez, Luis Alejandro Perez-Ruano, Jose A Figueroa-Sanchez

**Affiliations:** 1 Neurosurgery, Tecnológico de Monterrey, Monterrey, MEX; 2 Medicine, Instituto de Neurología y Neurocirugía, Hospital Zambrano Hellion TecSalud, San Pedro Garza García, MEX; 3 Medicine, Tecnológico de Monterrey, Monterrey, MEX; 4 Radiology, Tecnologico de Monterrey, Monterrey, MEX; 5 Neurological Surgery, Tecnologico de Monterrey, Monterrey, MEX

**Keywords:** categorization, classification, fistula, leak, csf, cerebrospinal fluid

## Abstract

Fistulas are abnormal communications between body cavities. They can occur between the CNS and the extracranial space, presenting clinically as CSF leaks. Due to the variety of features, multiple classifications have been implemented to better study this pathology.

A systematic review was conducted using the Scopus, Medline, and Web of Science databases. Observational studies such as cohort studies, case reports, case series, cross-sectional studies, systematic reviews, and publications that assess the classification of CSF leaks were included.

The systematic review identified 29 publications that met the required criteria for inclusion. Although the primary focus of most of these publications was not on classification, they briefly mentioned it. The included publications describe classifications according to etiology, exiting flow pressure, anatomic site, and some new classification proposals. Of the 29 included studies, 11 referred to the appearance of CSF rhinorrhea or otorrhea with no relationship between the cause or site of origin and the site of the CSF leak. However, none of these publications names this situation. These results clearly indicate that a term for this circumstance needs to be established; none of the previously listed publications provide a name for this condition.

This systematic review aims to demonstrate the necessity of implementing a new term to describe CSF leaks where the 'apparent origin' does not correspond to the 'real origin.' The results show no existing term that considers such cases; therefore, we propose the term 'Indirect Fistula' to designate these cases.

## Introduction and background

The term 'CSF leak' or 'CSF fistula' refers to an abnormal connection between the subarachnoid space and the external space that allows CSF communication [1]. A leak occurs when a dural tear or defect induces a CSF shift from the subarachnoid space into the extracranial space, allowing CSF to flow along a pressure gradient, thus giving rise to a fistula [2,3]. 

The description and classification of CSF leaks have remained the same since their first description by Ommaya AK et al. in 1960, who proposed the most widely accepted classification to date. It is a simple division of traumatic and non-traumatic or spontaneous, with the former caused by a head injury or iatrogenically [3-6]. St. Clair Thompson initially proposed the term 'rhinorrhea' in 1899 [3]. He compiled a series of patients with nasal CSF leaks and coined the term rhinorrhea [3]. Due to the short survival rates of patients who sustained severe head traumas that tore the dura, the leaks in these early series were of non-traumatic etiology [7].

CSF leaks are a common pathology, but a precise estimate of prevalence is uncertain due to the multiplicity of potential underlying causes and the underreported cases that are self-limited [8]. Approximately 40 to 80 percent of CSF leaks are secondary to head traumas [9-12]. Elective neurosurgery procedures might also result in the same complication, occurring in 3.8% of cases [13]. About 4% of CSF leaks are caused by various etiologies, including congenital abnormalities and spontaneous leaks [14,12]. The latter is associated with female gender, obesity, and obstructive sleep apnea [15].

The clinical features of CSF fistulas vary depending on the site of the leak and the amount of CSF lost [1]. A fistula between the dura and the anterior base of the skull most commonly causes CSF rhinorrhea, presenting as a hyaline discharge from the nose [1]. Intracranial volume and pressure from this extradural CSF leaking may cause neurological symptoms, such as headache, tinnitus, hearing loss, aural fullness, pneumocephalus, meningitis, or a brain abscess [16]. In the case of uncorrected CSF leaks, mortality has been documented to be 9% after one year [17]. Diagnosing this condition requires a thorough physical examination, dedicated imaging, and laboratory testing [1].

When a clear nasal discharge is present, CSF markers should be analyzed to confirm the presence of CSF [4]. Localization of the leak site involves radiological investigation, mainly through CT and MRI, to find the exact location of the dural tear and ensure successful obliteration of the fistula [18].

However, there are circumstances where a dural tear occurs, mainly postoperative or congenital, and the CSF exits at a distinct (or remote) cranial site. We describe one case, previously published by the senior author, as an example of a remote dural tear and CSF fistula, where the CSF leak presents as rhinorrhea even though the dural tear is not related to any paranasal sinus. Therefore, we propose the introduction of 'Indirect Fistula' as a new term to describe such cases.

## Review

Material and methods

**Figure 1:**
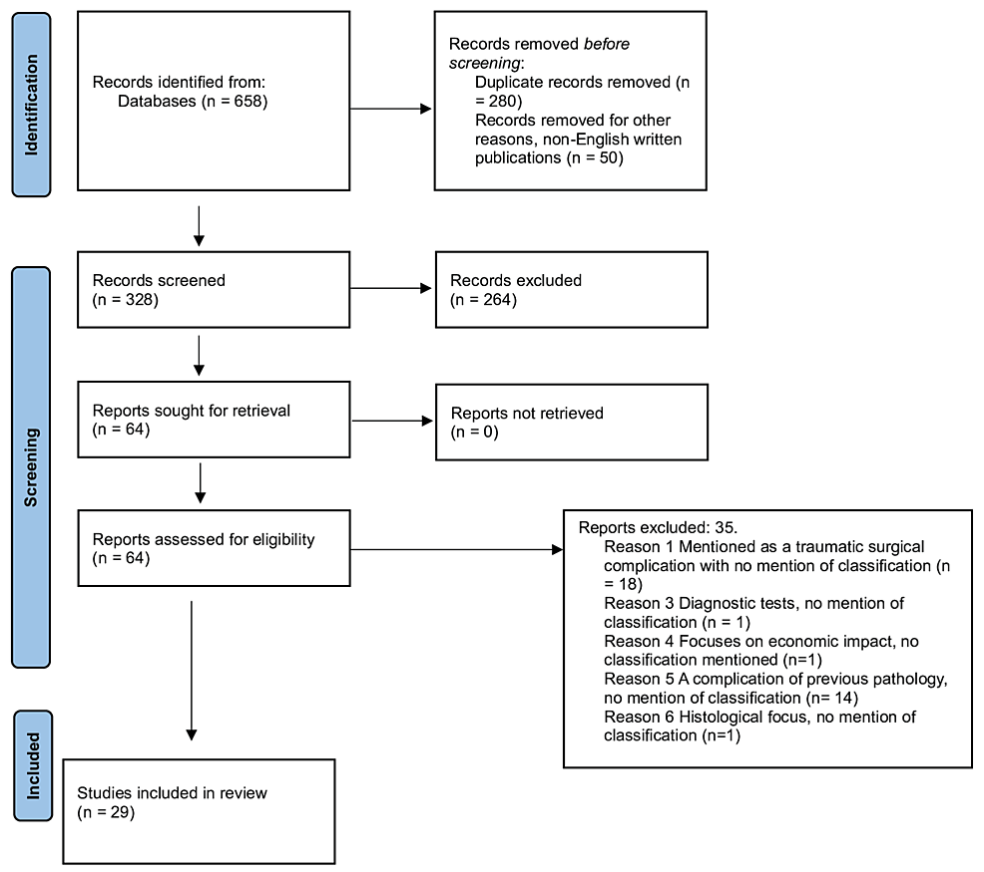
Preferred Reporting Items for Systematic Review and Meta-Analysis (PRISMA) 2020 flow diagram for systematic reviews.

The protocol described was performed in compliance with the Preferred Reporting Items for Systematic Reviews and Meta-Analyses (PRISMA) 2020 guidelines. The literature search was conducted to review the various types of CSF leak classifications using selected databases: Scopus, Medline, and Web of Science (Figure 1).

Search Strategy 

To gather information for this review, an unrestricted search was performed in May 2023, limited to Scopus, Medline, and Web of Science. No time period filter was applied; all results up to May 2023 were included. We developed search strategies using the keywords "cerebrospinal fluid" or "CSF," "leak or fistula," and "classification" or "categorization." The search was limited to the titles, abstracts, and keywords of scientific articles.

Search Validation and Data Selection 

Using the search criteria, all relevant publications were located, and those accessible on the specified databases at the time of this review were included. Citations were downloaded to a reference management software, and duplicate articles were manually removed. At this stage, the selected papers were screened to find those that fulfilled the inclusion criteria. For these articles, the full text was obtained and reviewed. Results were extracted by Carlos D. Acevedo-Castillo and Luis Alejandro Perez-Ruano, and disagreements about any specific included or omitted study were settled by a senior neurosurgeon (Enrique Caro-Osorio). Subsequently, the studies reporting various types of CSF leak categorization were evaluated.

*Inclusion Criteria* 

Eligibility was limited to publications that were observational studies, such as cohort studies, case series, cross-sectional studies, systematic reviews, and case reports, due to the limited representation of cases. Publications considered mentioned a type of classification for CSF leaks. Additionally, we included research in which indirect pathways caused the appearance of CSF rhinorrhea or otorrhea. Furthermore, we reviewed articles and textbooks that provided further information about CSF leaks to complement the traditionally used etiological classification.

Description of Study Selection

In our initial search, 658 studies were found. After eliminating duplicates, 378 references remained. Of these, 50 articles in languages other than English were eliminated, leaving 328 articles. After an initial examination of titles and abstracts, 64 citations were chosen for full-text reviews. Thirty-five studies were disregarded because there was no information on the classification of CSF leaks or the appearance of non-direct pathways of these leaks. Finally, 29 manuscripts were included in this evaluation. In addition, various related literature articles and book chapters considered relevant to the subject were manually included, mentioning the most common classifications.

Quality Assessment

We used JBI's critical appraisal tools to assess the included studies, which were Case Series, Case Reports, Systematic Reviews, Research Syntheses, and Cohort studies [19-21]. We assessed the papers' trustworthiness, relevance, and results.

Characteristics of Studies 

The 29 studies included in the systematic search were published between June 1968 and May 2023. Most were conducted in the United States. In addition to these, 12 review articles and two book chapters were added as reference material.

Results 

We identified 29 publications that met the required criteria for inclusion (Table 1). Although most of these publications did not focus primarily on classification, they briefly mentioned it. Of the 29 included studies, 11 refer to the appearance of CSF rhinorrhea or otorrhea with no relationship between the apparent and real origin, but none give this situation a proper name.

**Table 1:** Included studies classification.

Number	Classification	Author	Title
1	Location	Schievink et al., 2021	Long-term Risks of Persistent Ventral Spinal CSF Leaks in SIH: Superficial Siderosis and Bibrachial Amyotrophy
2	Location	Beck et al., 2019	Posterior Approach and Spinal Cord Release for 360° Repair of Dural Defects in Spontaneous Intracranial Hypotension
3	Location	Farnsworth et al., 2023	Spontaneous intracranial hypotension: Updates from diagnosis to treatment
4	Flow-rate	Cong et al., 2019	Simple dural closure using a knotless barbed suture in endoscopic transsphenoidal surgery: preliminary experience
5	Flow-rate	Park et al., 2015	Modified Graded Repair of Cerebrospinal Fluid Leaks in Endoscopic Endonasal Transsphenoidal Surgery
6	Flow-rate	Thomas et al., 2016	Principles in Skull Base Reconstruction following Expanded Endoscopic Approaches
7	Dura matter rupture anatomy	Galarza et al., 2018	Evaluation and management of small dural tears in primary lumbar spinal decompression and discectomy surgery
8	Etiology (no correlation of “real” and “apparent” site of leak)	Perry et al., 2019	Little Insights from Big Data: Cerebrospinal Fluid Leak After Skull Base Surgery and the Limitations of Database Research
9	Etiology (no correlation of “real” and “apparent” site of leak)	Alattar et al., 2018	Risk Factors for Readmission with Cerebrospinal Fluid Leakage Within 30 Days of Vestibular Schwannoma Surgery
10	Etiology (no correlation of “real” and “apparent” site of leak)	Kaufman et al., 1979	Acquired middle cranial fossa fistulas: normal pressure and nontraumatic in origin
11	Etiology (no correlation of “real” and “apparent” site of leak)	Wilson et al., 2014	The Management of Spontaneous Otogenic CSF Leaks: A Presentation of Cases and Review of Literature
12	Etiology (no correlation of “real” and “apparent” site of leak)	Yang et al., 2022	Petrous bone cholesteatoma presenting as CSF rhinorrhea: An extremely rare case report
13	Etiology (no correlation of “real” and “apparent” site of leak)	Ommaya et al., 1968	Non-traumatic cerebrospinal fluid rhinorrhoea
14	Etiology (no correlation of “real” and “apparent” site of leak)	Liang et al., 2001	Non-traumatic cerebrospinal fluid rhinorrhea indirectly caused by remote brain tumor: a case report and review of the literature.
15	Etiology (no correlation of “real” and “apparent” site of leak)	Calcaterra et al., 1977	Cerebrospinal rhinorrhea: extracranial surgical repair.
16	Etiology (no correlation of “real” and “apparent” site of leak)	Johnson et al., 2008	Temporal bone fracture: evaluation and management in the modern era
17	Etiology (no correlation of “real” and “apparent” site of leak)	Phang et.al., 2016	Management of CSF leak in base of skull fractures in adults.
18	Etiology (no correlation of “real” and “apparent” site of leak)	Sivanandapanicker et al., 2018	Analysis and Clinical Importance of Skull Base Fractures in Adult Patients with Traumatic Brain Injury
19	Etiology	Har-El, 1999	What is “Spontaneous” Cerebrospinal Fluid Rhinorrhea? Classification of Cerebrospinal Fluid Leaks.
20	Etiology	Oh et al., 2017	Traumatic Cerebrospinal Fluid Leak: Diagnosis and Management.
21	Etiology	Zlab et al., 1992	Cerebrospinal fluid rhinorrhea: a review of the literature
22	Etiology	Zahedi et al., 2022	Management of Traumatic and Non-Traumatic Cerebrospinal Fluid Rhinorrhea-Experience from Three Southeast Asian Countries
23	Etiology	Alonso et al., 2013	Spontaneous skull base meningoencephaloceles and cerebrospinal fluid fistulas
24	Etiology	Baban et al., 2017	Radiological and clinical interpretation of the patients with CSF leaks developed during or after endoscopic sinus surgery
25	Etiology	Coucke et al., 2022	The incidence of postoperative cerebrospinal fluid leakage after elective cranial surgery: a systematic review
26	Etiology	Le et al., 2016	Management of Anterior Skull Base Cerebrospinal Fluid Leaks
27	Etiology	Petri et al., 2018	Horizontal lateral lamella as a risk factor for iatrogenic cerebrospinal fluid leak
28	Etiology	Felisati et al., 2008	Italian multicentre study on intrathecal fluorescein for craniosinusal fistulae
29	Etiology	Daele et., al 2011	Traumatic, iatrogenic, and spontaneous cerebrospinal fluid (CSF) leak: endoscopic repair

Eleven of the included papers use etiology as a classification system, covering commonly known leak causes such as traumatic, non-traumatic, iatrogenic, and spontaneous [6,7,9,10,11,13,14,18,22-24].

Three studies categorized leaks based on location and were used for spinal cord dural defects, dividing them into ventral (anterior dural sac), lateral (including nerve root exit, lateral, and dorsal dural sac), and foraminal [25-27]. Three publications used Kelly's 4-grade classification system of CSF leaks according to flow pressure, which can be graded from 0 (no flow) to 3 (high flow); one of these proposed a modified intraoperative system [28-30].

We also found a published case study in which the authors used an anatomical classification based on the internal characteristics of the dura mater rupture rather than the anatomical location of the durotomy or the vertebral location [31]. This description is proposed for dural tears smaller than 1 cm [31].

A few articles, including Ommaya AK's classification, mention a type of fistula originating from a tumor whose location does not correlate with the 'apparent site' of the leak [5]. One article describes non-traumatic CSF rhinorrhea as an indirect manifestation of remote brain tumors [32]. The cases were divided into categories due to feature variations [32]. As in the previous case, nine more studies mention representative cases in which various causes are clinically present, such as CSF rhinorrhea or otorrhea, with no correlation of the dural tear with the apparent origin. Some are attributed to traumatic events, including fractures of the mid-skull base [33-36]. Two are postoperative complications [37,38]. The last three are classified as non-traumatic causes [39-41].

**Figure 2:**
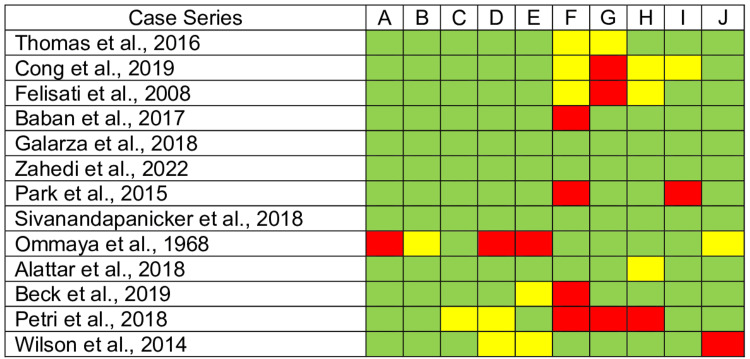
Critical appraisal tools for case series. Green: Yes, Red: No, Yellow: Unclear. A. Were there clear criteria for inclusion in the case series? B. Was the condition measured in a standard, reliable way for all participants included in the case series? C. Were valid methods used for the identification of the condition for all participants included in the case series? D. Did the case series include participants consecutively? E. Did the case series include all participants completely? F. Was there clear reporting of the demographics of the participants in the study? G. Was there clear reporting of the clinical information of the participants? H. Were the outcomes or follow-up results of the cases clearly reported? I. Was there clear reporting of the presenting site(s)/clinic(s) demographic information? J. Was the statistical analysis appropriate?

**Figure 3:**
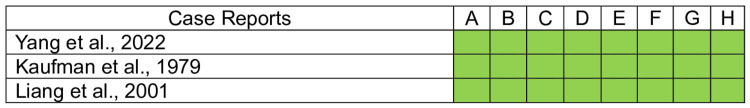
Critical appraisal tools for case reports. Green: Yes, Red: No, Yellow: Unclear. A. Were the patient’s demographic characteristics clearly described? B. Was the patient’s history clearly described and presented as a timeline? C. Was the patient’s clinical condition upon presentation clearly described? D. Were the diagnostic tests or assessment methods and their results clearly described? E. Were the interventions or treatment procedures clearly described? F. Was the patient’s clinical condition after the intervention clearly described? G. Were any adverse events (harms) or unanticipated events identified and described? H. Does the case report provide takeaway lessons?

**Figure 4:**
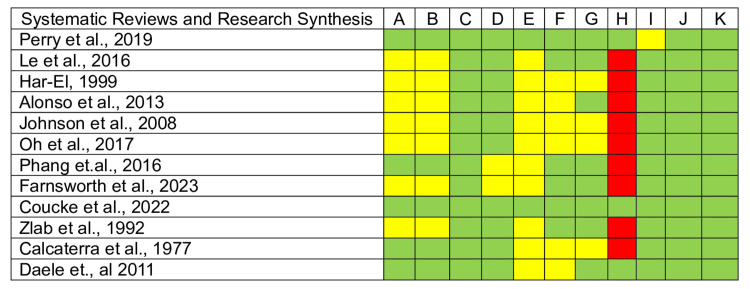
Critical appraisal tools for systematic reviews and research synthesis. Green: Yes, Red: No, Yellow: Unclear. A. Was the review question clearly and explicitly stated? B. Were the inclusion criteria appropriate for the review question? C. Was the search strategy appropriate? D. Were the sources and resources used to search for studies adequate? E. Were the criteria for appraising studies appropriate? F. Was critical appraisal conducted by two or more reviewers independently? G. Were there methods to minimize errors in data extraction? H. Were the methods used to combine studies appropriate? I. Was the likelihood of publication bias assessed? J. Were the recommendations for policy and/or practice supported by the reported data? K. Were the specific directives for new research appropriate?

**Figure 5:**
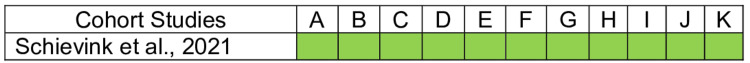
Critical appraisal tools for cohort studies. Green: Yes, Red: No, Yellow: Unclear. A. Were the two groups similar and recruited from the same population? B. Were the exposures measured similarly to assign people to both exposed and unexposed groups? C. Was the exposure measured in a valid and reliable way? D. Were confounding factors identified? E. Were strategies to deal with confounding factors stated? F. Were the groups/participants free of the outcome at the start of the study (or at the moment of exposure)? G. Were the outcomes measured in a valid and reliable way? H. Was the follow up time reported and sufficient to be long enough for outcomes to occur? I. Was follow up complete, and if not, were the reasons to loss to follow up described and explored? J. Were strategies to address incomplete follow up utilized? K. Was appropriate statistical analysis used?

Finally, the quality assessment results show that the included studies are trustworthy and relevant. The case series and case reports included comply with the critical appraisal checklist (Figures 2-3). However, systematic reviews and research syntheses lack a publication bias assessment (Figure 4). Moreover, the case series need clearer reporting of participants' demographics, clinical information, and outcomes (Figure 5). Altogether, all articles included have an adequate overall appraisal.

Representative Case

This case was previously reported by the senior author [42]. A 34-year-old female patient was admitted to the hospital with vomiting, headache, and deteriorating consciousness. She had a 2-year history of "intermittent right unilateral hyaline rhinorrhea." The leak originated from the cribriform plate to the right ethmoid sinus, according to initial CT cisternography (CTC) results. During an endoscopic examination of the nasal cavity using fluorescein, a leak from the right Eustachian tube was discovered, with no damage to the ethmoid roof [42].

A second CTC examination raised the possibility of a dehiscence in the middle fossa floor communicating with the oval window. Consequently, a right temporal craniotomy was conducted, detaching some dural adhesions from the floor with cribriform-like structures and covering it with temporal fascia [42].

Due to persistent symptoms, contrast-enhanced magnetic resonance cisternography (CE-MRC) was performed. It showed contrast material extending from the right internal auditory canal to the inner and middle ear along a path near the oval window. A CT scan of the temporal bones demonstrated an incomplete partition type II anomaly of the right cochlear apex and an enlarged vestibular aqueduct consistent with Mondini deformity. A petrosectomy with occlusion of the defect was performed, and the fistula finally resolved [42].

The approach consisted of reopening the craniotomy and localizing the dural defect with the help of CE-MRC and neuronavigation. A dural dehiscence of approximately 15 mm was found, extending below the bone, accompanied by CSF leakage in the frontal region. The autologous galea patch was sutured and secured at the dural tear. In addition, cranialization and frontal sinus obliteration were performed, achieving definitive control of the fistula [42].

Discussion

CSF leak is a pathology capable of causing multiple complications [5]. Since its first description, several classifications have been implemented to better describe the different varieties of presentation [8]. We identified 29 studies mentioning different classifications used to characterize CSF leaks. As previously mentioned, the most common and straightforward classification system used is according to etiology, dividing it into traumatic and non-traumatic, as per Ommaya AK et al. [5].

Traumatic CSF fistulas can be accidental, occurring most frequently from blunt trauma leading to head injuries and fractures of the skull base. These can be subclassified as penetrating and non-penetrating [6,43]. Traumatic leaks can also originate from iatrogenic complications of endoscopic sinus surgery, endoscopic endonasal transsphenoidal tumor surgery, and lateral skull base surgery [23,25,29,36].

There are two types of nontraumatic CSF leaks: high-pressure and normal-pressure [5]. The former can be a manifestation of tumors, hydrocephalus, or idiopathic intracranial hypertension. In Ommaya AK's original description of CSF leaks, he mentions that a tumor in a remote location can give rise to a CSF fistula where its "real" and "apparent" origin is at the anterior skull base, consequently causing rhinorrhea [5]. This is due to an increase in intracranial pressure, not the tumor itself [5]. Considering our concept, this situation would be considered a type of Direct Fistula.

Normal pressure leaks usually occur due to arachnoid granulations, idiopathically, infections, empty sella, or congenital causes [39]. These fistulas can also present as Indirect, with the dural defect distal to the apparent origin, as in the representative case [44]. Other congenital causes may involve the failure of closure of the anterior neuropore, leading to herniation of the meninges and brain through the defect, or a persistent cricopharyngeal canal [45].

Most fistulas have direct communication between the dural tear and the apparent site of leaking, forming their respective trajectories as real fistulas [16]. In these cases, we find the attachment of the dural tear at the bone defect, and in others, herniation of the brain parenchyma may be observed [18]. This is what we refer to as a "Direct Fistula."

However, there is a percentage of cases in which patients present with atypical clinical presentations and uncertain imaging features, where the clinician is still unsure of the actual site of the dural tear that gives origin to the CSF leak [24,46]. When clinical suspicion persists and there is a lack of proof provided by essential imaging studies where the "leaking" of CSF is not occurring in direct connection with the "real origin" or dural tear, a more thorough imaging examination, such as CE-MRC, should be carried out [42].

As far as we know, no term considers a dural tear that gives rise to a fistula through a distal exiting cranial site, such as the representative example presented and the cases mentioned in the articles included in our systematic review. We propose referring to the exiting cranial site of CSF as "apparent" and the actual site of the dural tear as "real," identifying these leaks as "Indirect Fistulas."

By having these two concepts clearly defined, it is possible to account for the many cases in which this situation occurs. Accurately identifying the exact location of the leak prior to surgery is crucial for the surgical approach and increases the likelihood that the procedure will be successful while reducing the risk of complications [42].

Ultimately, it establishes a clear guide for approach and treatment, which orients the clinician in using the proper evaluation techniques, such as the case of CE-MRC. This allows the timely diagnosis of indirect fistulas and provides a definitive surgical solution.

Limitations 

We used a thorough search strategy by critical review tasks to find all the papers that evaluated the categories of CSF leaks for this investigation. Even so, we likely overlooked unpublished data, as we did not explore all databases or grey literature, although we observed no publishing bias. Additionally, the study was not registered with PROSPERO, although a search for similar publications was performed with no relevant results found. Finally, we believe that a larger group of representative cases is needed to modify the established general classification.

## Conclusions

The fistula is an abnormal connection between the subarachnoid and external space, allowing CSF communication. Not all fistulas are direct; sometimes, they have a distant origin from the apparent leak. Even though there are many proposed classifications for CSF leaks in the literature, there is no term to allude to a dural tear giving origin to a fistula through a distal exiting cranial site. In these cases, it seems reasonable to use the term Indirect Fistula. A thorough search, such as a CE-MRC or CISS MRI sequence, is advised under these circumstances. This will enable an adequate and timely diagnosis using the proper tools, providing a resolutive treatment.
